# Influence of synthesis conditions on microstructure and phase transformations of annealed Sr_2_FeMoO_6−x_ nanopowders formed by the citrate–gel method

**DOI:** 10.3762/bjnano.7.111

**Published:** 2016-08-22

**Authors:** Marta Yarmolich, Nikolai Kalanda, Sergey Demyanov, Herman Terryn, Jon Ustarroz, Maksim Silibin, Gennadii Gorokh

**Affiliations:** 1 Scientific-Practical Materials Research Centre, NAS of Belarus, Minsk 220072, Belarus; 2 National Research University of Electronic Technology “MIET”, Moscow 124498, Russia; 3 Research Group Electrochemical and Surface Engineering, Vrije Universiteit Brussel, Brussel 1050, Belgium; 4 Belarusian State University of Informatics and Radioelectronics, Minsk 220013, Belarus,; 5 ITMO University, St. Petersburg 197101, Russia

**Keywords:** magnetic materials, microstructure, nanoparticles, phase transformation, sol–gel preparation

## Abstract

The sequence of phase transformations during Sr_2_FeMoO_6−x_ crystallization by the citrate–gel method was studied for powders synthesized with initial reagent solutions with pH values of 4, 6 and 9. Scanning electron microscopy revealed that the as-produced and annealed powders had the largest Sr_2_FeMoO_6−x_ agglomerates with diameters in the range of 0.7–1.2 µm. The average grain size of the powders in the dispersion grows from 250 to 550 nm with increasing pH value. The X-ray diffraction analysis of the powders annealed at different temperatures between 770 and 1270 K showed that the composition of the initially formed Sr_2_FeMoO_6−x_ changes and the molybdenum content increases with further heating. This leads to a change in the Sr_2_FeMoO_6−x_ crystal lattice parameters and a contraction of the cell volume. An optimized synthesis procedure based on an initial solution of pH 4 allowed a single-phase Sr_2_FeMoO_6−x_ compound to be obtained with a grain size in the range of 50–120 nm and a superstructural ordering of iron and molybdenum cations of 88%.

## Introduction

Due to their unique and extremely important magneto-transport and magnetic properties [1–2], metal oxide Sr_2_FeMoO_6−x_ systems with an ordered double perovskite structure are among the most promising materials for spintronic devices [3–6]. However, the synthesis of strontium ferromolybdate by conventional methods [2–3,7–8], including solid-state synthesis using high-temperature annealing in a reducing atmosphere with predetermined anionic and cationic defectiveness, is problematic [9]. This is due to several factors: the phase purity within the sample, cation and anion vacancies, sample microstructure, chemical composition and thickness of the grain boundaries [10–13]. Sol–gel technology is a relatively new, but very promising method to synthesize nanoscale, double perovskite materials at relatively low temperatures and in a shorter time [3–4,14]. However, despite numerous articles devoted to this subject [3,7–8,15–16], the optimization of a sol–gel-based synthesis procedure to obtain nanoscale single-phase Sr_2_FeMoO_6−x_ with a maximum degree of superstructural ordering, still remains a challenge. The main problem is the formation of secondary phases such as Fe, Sr_3_MoO_6_, Fe_3_O_4_, and SrMoO_4_, the latter practically impossible to remove [3,7–8,10,14,16]. Moreover, increasing the synthesis temperature improves the superstructural ordering of Fe/Mo cations but at the same time leads to an increase of the grain size of the Sr_2_FeMoO_6−x_ phase [3,7–8,11,17]. On the one hand, it is possible to obtain a single phase powder, but on the other hand, the grain size increases up to 500–800 nm [7–8,18].

Hence, the current state-of-the-art does not establish an accurate citrate–gel synthesis procedure to obtain a single-phase Sr_2_FeMoO_6−x_ nanoscale powder with a high degree of superstructural ordering and with optimal magnetic properties. In this regard, in the present work, we investigate the correlation between the citrate–gel synthesis conditions (pH of initial solutions and annealing temperature) and the microstructure and phase transformations of the Sr_2_FeMoO_6−x_ nanopowders.

## Results and Discussion

**Figure 1:**
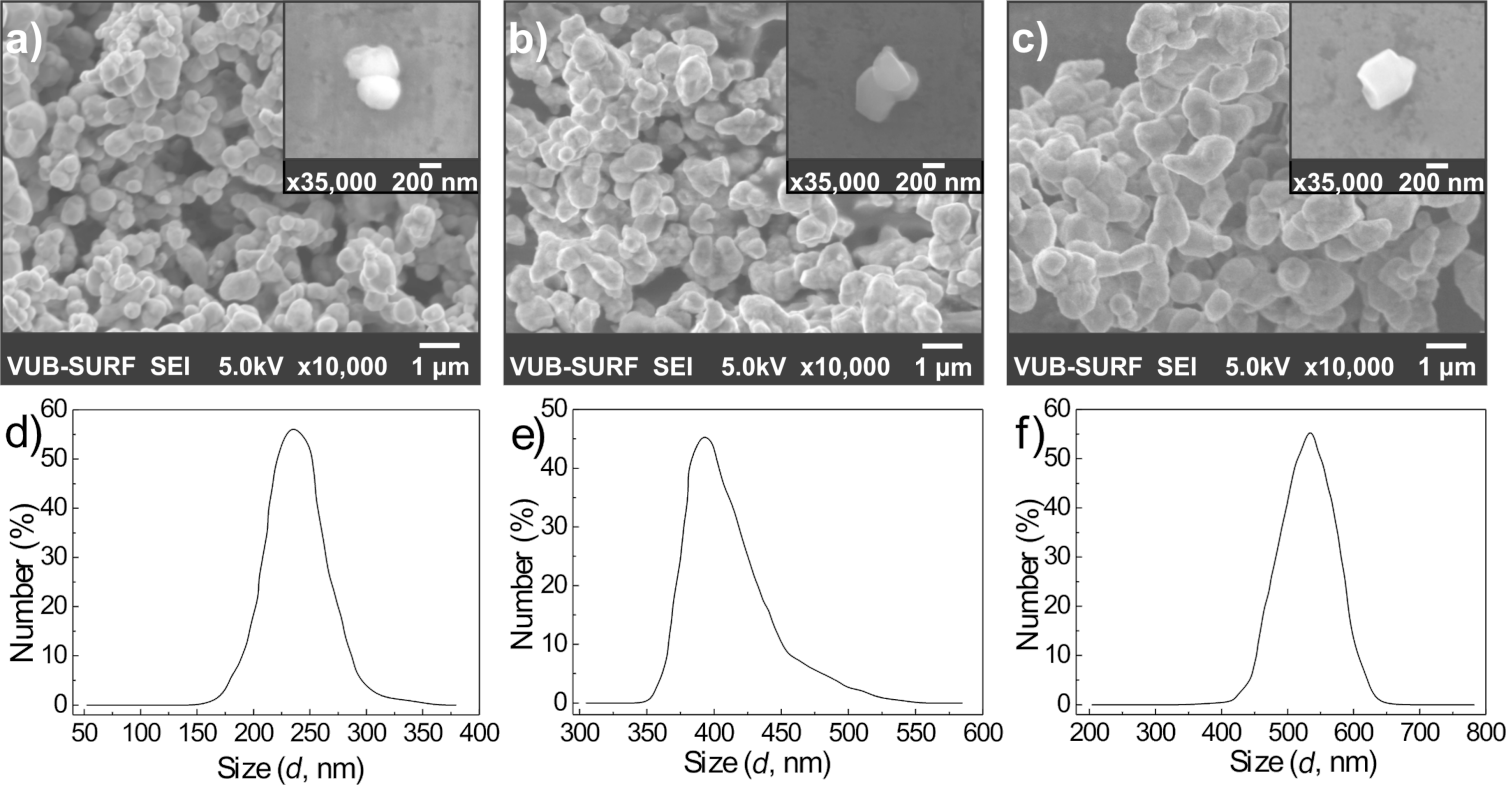
Field emission scanning electron microscopy (a–c) and atomic force microscopy images (insets in a–c) and particle size (diameter) distributions (d–f) obtained by dynamic light scattering of SFMO-4 (a,d), SFMO-6 (b,e), SFMO-9 (c,f) powder samples. The samples were annealed in the polythermal regime over a 770–1220 K temperature range, at 2 K/min, in a continuous stream of 5% H_2_/Ar gas mixture.

Figure 1a–c shows representative field emission scanning electron microscopy (FESEM) images of the microstructure of three Sr_2_FeMoO_6−x_ (SFMO) powder samples, SFMO-4, SFMO-6, and SFMO-9 (where the number indicates the pH), annealed in the temperature range of 770–1220 K at a heating rate 2 K/min in a continuous stream of 5% H_2_/Ar gas mixture. These images reveal the presence of large Sr_2_FeMoO_6−x_ agglomerates ranging in diameter from 0.7 to 1.2 µm. To obtain more information about the microstructural characteristics of the SFMO-4, SFMO-6, and SFMO-9 powder samples, dynamic light scattering (DLS) was also carried out. For these measurements, 10 mg of SFMO-4, SFMO-6, or SFMO-9 was dispersed in 20 mL of ethanol during 20 min. The particle size histograms (Figure 1d–f) obtained by DLS indicate that the average grain diameter of all the powders is between 250 and 550 nm. It becomes clear that the pH of the initial solutions influences the microstructure of the resulting Sr_2_FeMoO_6−x_ material. Increasing the pH leads to the growth of grain sizes from 150 to 650 nm. In the case of SFMO-4 (the lowest pH), grains down to 150–350 nm were obtained, as displayed in Figure 1d. Despite the agglomeration that occurs upon drying, the effect of pH is also visible in the FESEM images, as SFMO-4 (Figure 1a) presents finer microstructured grain than SFMO-9 (Figure 1c).

**Figure 2:**
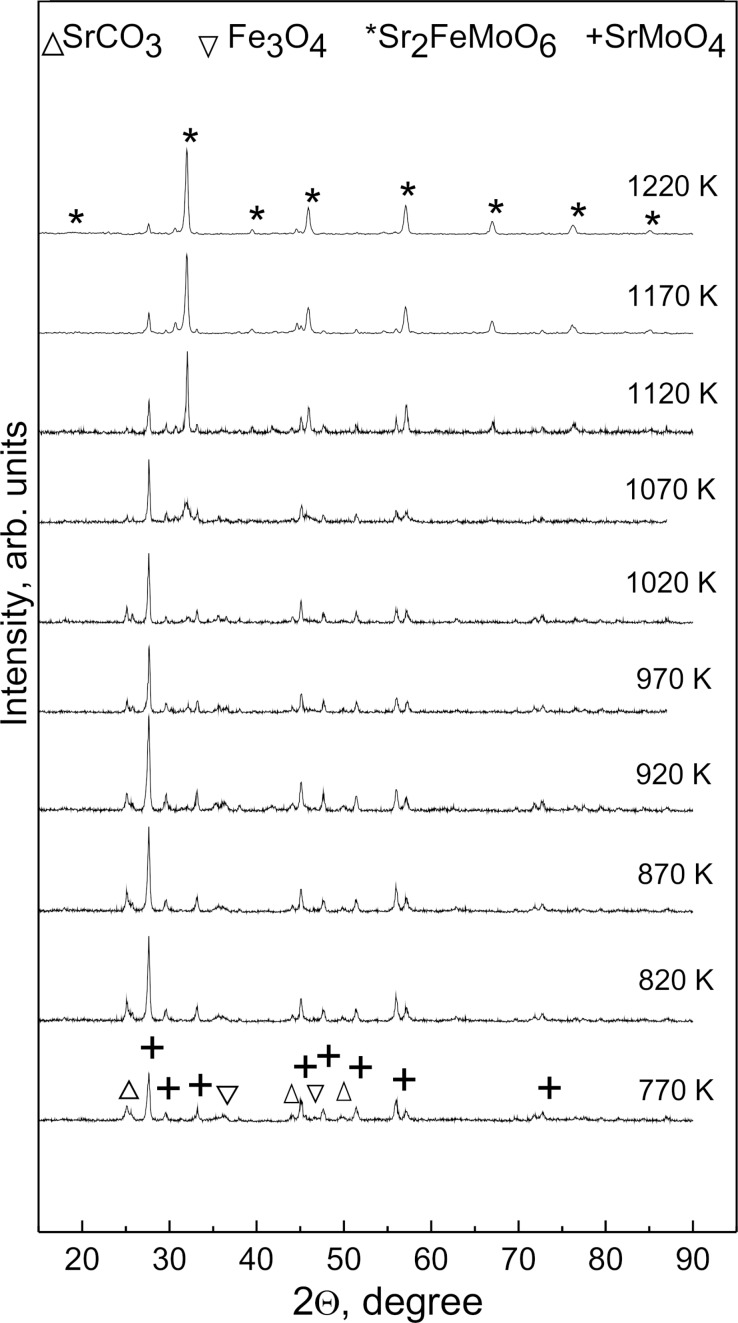
XRD patterns of the SFMO-9 powder sample annealed at 770–1270 K with a 2 K/min heat rate in a continuous stream of 5% H_2_/Ar gas mixture.

Figure 2 shows X-ray diffraction (XRD) diffractograms of the SFMO-9 powder sample at various stages of the synthesis at different temperatures. In order to study the sequence of phase transformations during Sr_2_FeMoO_6−x_ synthesis, SFMO-4, SFMO-6, and SFMO-9 powders were annealed in a continuous stream of 5% H_2_/Ar gas mixture between 770 and 1270 K, with a 2 K/min heating rate (temperature step 50 K). The evolution of the XRD diffractograms with temperature for the samples was similar, so only SFMO-9 is shown and can be considered representative, independent of the pH of the initial solutions. It can be seen that the synthesis of the solid solution of strontium ferromolybdate proceeds through a number of parallel chemical reactions that leads to several phase transformation processes.

It can be seen in the XRD data presented in Figure 2 that during the heating process that eventually leads to the formation of Sr_2_FeMoO_6−x_, several secondary phases are formed as well, such as SrMoO_4_, SrCO_3_ and Fe_3_O_4_. It is also shown that the amount of Sr_2_FeMoO_6−x_ as a solid solution increases as temperature increases, whereas the percentage of SrMoO_4_, SrCO_3_ and Fe_3_O_4_ phases decreases unequally. Eventually, at 1220 K, an almost single-phase strontium ferromolybdate compound with minimal content of SrMoO_4_ is observed.

**Figure 3:**
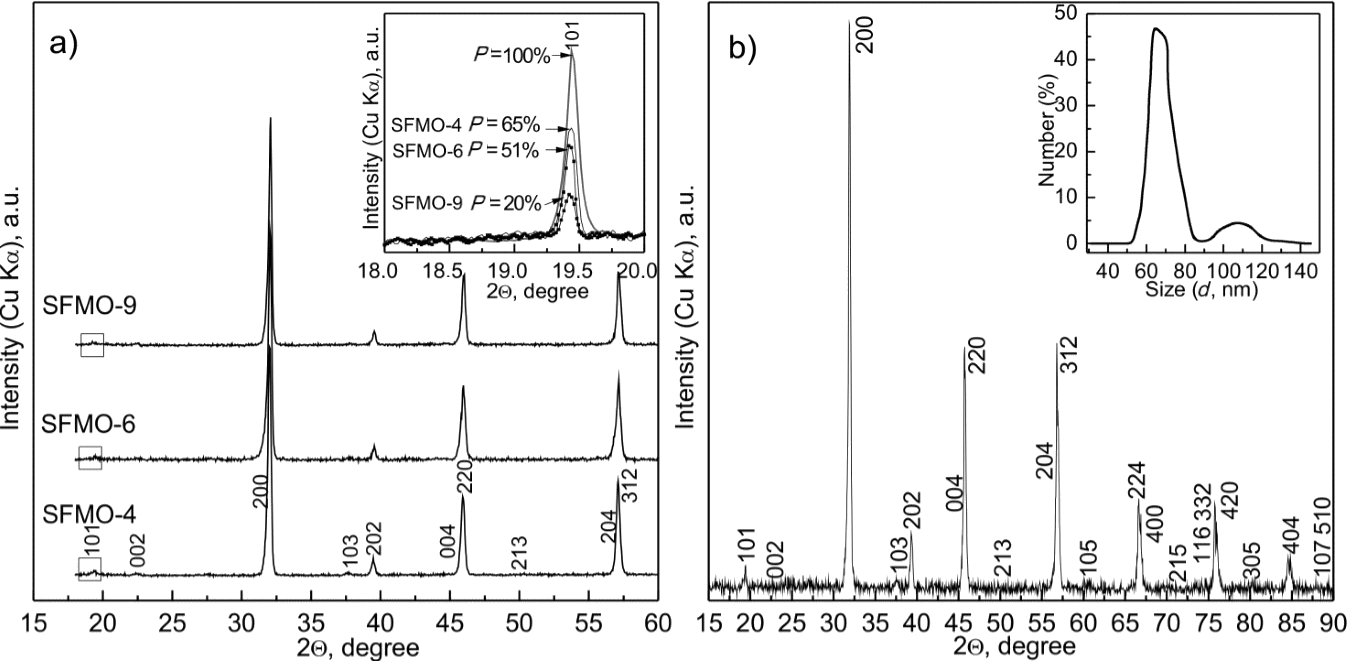
(a) XRD patterns of SFMO-4, SFMO-6, SFMO-9 power samples annealed at 1220 K in a continuous stream of 5% H_2_/Ar gas mixture at 2 K/min. Inset: Estimation of superstructural ordering degree of the iron and molybdenum cations. (b) X-ray diffractogram of Sr_2_FeMoO_6−x_ synthesized from a colloidal solution at pH 4 with varied annealing conditions (893 K for 1 h, 1060 K for 1 h and 1120 K for 4 h) and hardened at room temperature. Inset: Particle size distribution obtained by DLS.

From the XRD analysis of powders of SFMO-4, SFMO-6, SFMO-9 annealed at 1220 K (Figure 3a), it was found that an increase in pH of the colloidal solution results in a decrease in the intensity of the reflex (101) while the reflexes (200) remain substantially unchanged. A detailed analysis of the data and calculations (see Inset in Figure 3a) showed that SFMO-4, SFMO-6, and SFMO-9 powders have different degrees of superstructural ordering of Fe/Mo cations (interposition of iron and molybdenum ions relative to each other) [19]. A decrease in the degree of ordering of the superstructure with increasing pH of the solution was observed.

**Figure 4:**
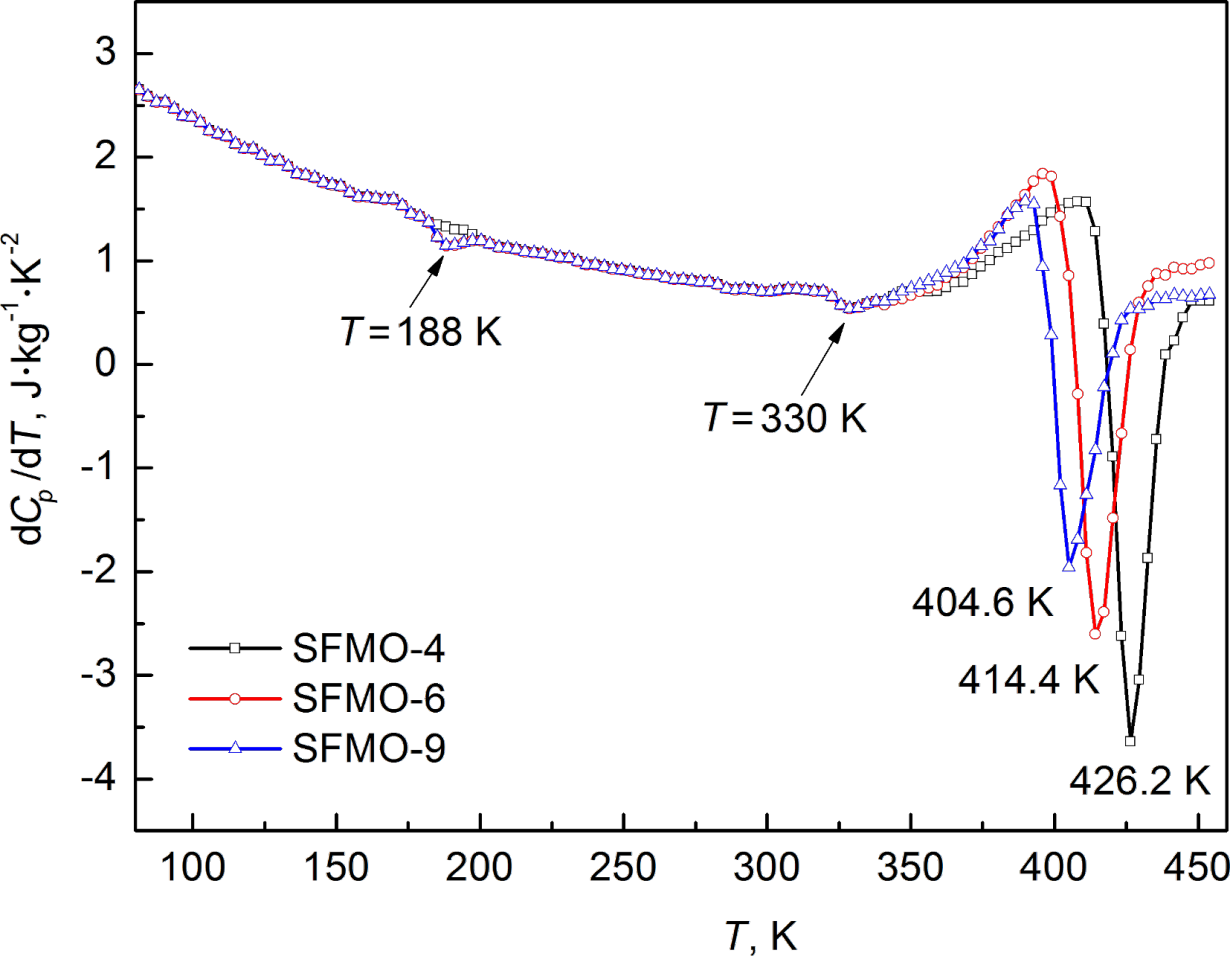
The temperature dependence of the heat capacity derivative of nanopowders, SFMO-4, SFMO-6 and SFMO-9.

Thus, the results of the X-ray analysis have shown that all investigated SFMO powders are single phase, although SFMO-4 should be distinguished, which has a higher degree of the superstructural ordering of Fe and Mo than the SFMO-6 and SFMO-9 powders. Nevertheless, according to the heat capacity temperature dependences (d*Cp*/d*T*) of the nanopowders, which were measured in the temperature range 120–450 K, the λ-type second-order phase transition has been revealed at 188 K for SFMO-6 and SFMO-9 powders and 330 K for all investigated powders (Figure 4). Such extremely small changes of entropy, as well as small temperature intervals of phase transformations (within 20 K), are caused by impurity phases distributed as inclusions in the basic, double perovskite matrix and magnetic phase transitions in local areas. The occurrence of this phase transition at 188 K in SFMO-6 and SFMO-9 powders could be related to the paramagnetic–antiferromagnetic, magnetic phase transition in the wustite (Fe_0.947_O) [20–22]. This indicates the non-single-phase nature of the SFMO-6 and SFMO-9 samples. Still, the λ-type second-order phase transition at 188 K has not been detected in the SFMO-4 powder, indicating that it has greater single-phase and magnetic homogeneity in comparison with the SFMO-6 and SFMO-9 samples. The heat capacity measurement has a higher sensitivity compared to the XRD investigations and makes it possible to determine the above-mentioned inclusions of the indirect phases with high reliability. In consideration of the highly λ-shaped transition at 330 K, we may presume that it is due to the transition of the antiferromagnetic inclusions in the ferromagnetic matrix to the paramagnetic state. The detected anomalies on the curves of the heat capacity temperature dependences are caused by the transformation temperature of about 426.2, 414.4, and 404.6 K, respectively. The temperature dependences of the magnetization [23] also confirm that assumption, which can be associated with the transition of Sr_2_FeMoO_6−x_ from the ferromagnetic to the paramagnetic state. It is noted that the dependence of the Curie temperature, *T*_C_, on the degree of the superstructural ordering has a complicated nonlinear appearance, and the *T*_C_ grows with the growth of the superstructural ordering of the investigated powders.

Table 1 shows the crystal lattice parameters and the degree of superstructural ordering of SFMO-4, SFMO-6, and SFMO-9 powder samples annealed at 1120, 1170, and 1220 K. These data revealed that the highest degree of ordering of the superstructure can reach a value of *P* = 65% in the powders obtained from solutions with pH 4.

**Table 1:** Crystal lattice parameters and degree of superstructural ordering of Fe/Mo cations of SFMO-4, SFMO-6, and SFMO-9 powder samples annealed for 4 h at 1120, 1170, and 1220 K.

Sample name	pH	*Т* [K]	*a* [Å]	*с* [Å]	*V* [Å^3^]	*P* [%]

SFMO-4	4	1120 1170 1220	5.5754 5.5691 5.5676	7.9003 7.8968 7.8911	245.581 244.918 244.609	56 60 65
SFMO-6	6	1120 1170 1220	5.5709 5.5692 5.5684	7.9035 7.8979 7.8939	245.284 244.961 244.766	44 50 51
SFMO-9	9	1120 1170 1220	5.5774 5.5722 5.5706	7.9087 7.9043 7.8991	246.019 245.423 245.121	12 17 20

Thus, the electron microscopy data and XRD analysis of SFMO-4, SFMO-6, and SFMO-9 powders have shown that the SFMO-4 powder had the smallest phase content of SrMoO_4_ (≈2%), the highest value of superstructural ordering of Fe/Mo cations (65%) and the smallest grain size (≈300–350 nm). Based on this, to obtain a more perfect single-phase powder of Sr_2_FeMoO_6−x_ with maximum superstructural ordering, the optimal route of a variable phase annealing of SFMO-4 powders was identified. Hence, to obtain a single-phase nanoscale Sr_2_FeMoO_6−x_ powder, combined heating steps are need at different stages of the annealing. During a preliminary synthesis, in the polythermal mode, the temperature should be raised at 2 K/min to *T* = 893 K and held constant for 1 h. To accelerate the decomposition of the SrMoO_4_ intermediate phase and to reach 100% of Sr_2_FeMoO_6−x_ phase transformation, the temperature should be raised up to *T* = 1060 K and held at this temperature for 1 h. The final Sr_2_FeMoO_6−x_ synthesis should be carried out at *T* = 1120 K for 4 h – precisely in these conditions, a single-phase powder is formed with nanoscale grains and superstructural ordering of Fe and Mo cations.

Using such a varied heating scheme, with an initial solution of pH 4, we succeeded in obtaining a single-phase Sr_2_FeMoO_6−x_ compound with lattice parameters *a* = *b* = 5.5629 Å, *c* = 7.8936 Å, *V* = 244.2742 Å^3^, having a grain size in the range of 50–120 nm and *P* = 88% (Figure 3b). To the best of our knowledge, typical superstructural orderings between 14–90% have been previously obtained [3,8,14]. Besides, a single-phase compound with a grain size of about 1–2 µm and with a superstructural ordering up to 95% was obtained at 1273 K [17]. However, in none of these cases was a single-phase material with small grain size obtained [3,7–9,14,24]. Hence, the synthesis procedure proposed in this work leads to Sr_2_FeMoO_6−x_ powders with enhanced properties and can thus be considered an improvement over the current cutting edge technology.

## Conclusion

The pH value of the colloidal solution and the annealing temperature of the powders have a significant impact on the microstructural properties of Sr_2_FeMoO_6−x_ prepared by the citrate–gel synthesis. The formation of a strontium ferromolybdate solid solution proceeds through a series of parallel chemical reactions with the formation of intermediate phases SrMoO_4_, SrCO_3_ and Fe_3_O_4_, and its relative amount is reduced with increasing temperature. This leads to an increased amount of Sr_2_FeMoO_6−x_ phase. Therefore, the lowest amount of strontium molybdate was observed for *T* = 1220 K, in the case where the initial solution had pH 4. With increasing temperature, the composition of strontium ferromolybdate changes and its molybdenum content increases. This leads to a change in the crystal lattice parameters and contraction of the unit cell. Based on the information obtained through the analysis of pH and temperature effect on the phase composition and microstructure, an improved procedure was designed based on an initial solution of pH 4. Single-phase Sr_2_FeMoO_6−x_ with a grain size in the range of 50–120 nm and a superstructural ordering of 88% was obtained. It was shown that by understanding its crystallization process, a Sr_2_FeMoO_6−x_ material with enhanced properties can be produced.

## Experimental

Sr_2_FeMoO_6−x_ powders were prepared by the citrate–gel route (a particular case of the sol–gel method), using Sr(NO_3_)_2_ (99.9%), Fe(NO_3_)_3_·9H_2_O (99.9%), (NH_4_)_6_Mo_7_O_24_ (99.9%) and citric acid monohydrate C_6_H_8_O_7_·H_2_O (99.9%) as starting materials. First, the aqueous solutions of 0.4 mol dm^−3^ Sr(NO_3_)_2_ and 2 mol dm^−3^ Fe(NO_3_)_3_ were mixed in a molar ratio of Sr/Fe 2:1, and then citric acid was added to the solution in a molar ratio of citric acid/Fe 6.5:1. After this, the 0.2 mol dm^−3^ (NH_4_)_6_Mo_7_O_24_ solution was added to the prepared aqueous Mo/Fe. By adding into three parts of this solution of ethylenediamine (EDA) the pH of the solutions were adjusted to values of 4, 6, and 9. Finally, the resulting solutions were continuously stirred at 350 K until the light green gels were formed. Heating of the gels was carried out at a rate of 0.4 K/min up to 470 K where the temperature was held for 18 h. The obtained solid foams were ground into powders and preheated at *T* = 770 K and pO_2_ = 0.21 × 10^5^ Pa in air for 10 h. Batches of annealed powders were identified accordingly as SFMO-4 (pH 4), SFMO-6 (pH 6) and SFMO-9 (pH 9) and then were used to study the sequence of phase transformations during Sr_2_FeMoO_6−x_ crystallization. In order to obtain a single-phase Sr_2_FeMoO_6−x_ powder, SFMO-4, SFMO-6 and SFMO-9 powders were annealed at temperatures ranging from 770 to 1270 K by means of a polythermic approach in a reducing atmosphere (5% H_2_/Ar gas mixture: 5% H_2_/95% Ar).

The microstructure of the SFMO powders was investigated using a JEOL JSM-7000F field emission scanning electron microscope. The grain size was evaluated using a NT-206 atomic force microscope. The particle size distributions of the SFMO powders were measured by dynamic light scattering using a Zetasizer Nano (Nano ZS90, Malvern, UK) particle analyser. The phase transformation degree (α, change of the phase content during synthesis process), lattice parameters and superstructural ordering degree of the calcined powders were determined by X-ray diffraction in a Siemens D5000 diffractometer with Cu Kα radiation (Bragg–Brentano para-focusing geometry and vertical θ–θ goniometer) equipped with a grazing incidence (ω = 1.7°) attachment for thin film analysis and a scintillation counter as a detector. The data were collected with an angular step of 0.05° at 5 s per step. The XRD patterns were reﬁned using the ICSD-PDF2 (Release 2000) database and the FULLPROF [19] and PowderCell [25] Rietveld reﬁnement programs.

The heat capacity of the nanopowders of SFMO-4, SFMO-6, and SFMO-9 were measured in the temperature range 120–450 K on the differential scanning calorimeter, DSC PT1000 produced by Linseis Messgeraete GmbH (Germany). The step of temperature rise in the measurement of the heat capacity was ≈2 K.
